# Alterations in meibomian glands in patients treated with intensity-modulated radiotherapy for head and neck cancer

**DOI:** 10.1038/s41598-021-01844-9

**Published:** 2021-11-17

**Authors:** Xiangjun Chen, Reza A. Badian, Håvard Hynne, Cecilie Delphin Amdal, Bente Brokstad Herlofson, Øygunn Aass Utheim, Kristine Løken Westgaard, Fredrik Fineide, Janicke Liaaen Jensen, Tor Paaske Utheim

**Affiliations:** 1 Department of Oral Surgery and Oral Medicine, Faculty of Dentistry, University of Oslo, Geitmyrsveien 71, 0455 Oslo, Norway; 2 The Norwegian Dry Eye Clinic, Oslo, Norway; 3 Department of Medical Biochemistry, Oslo University Hospital, Oslo, Norway; 4 Department of Oncology, Oslo University Hospital, Oslo, Norway; 5 Department of Ophthalmology, Oslo University Hospital, Oslo, Norway; 6 Department of Oral Biology, University of Oslo, Oslo, Norway

**Keywords:** Eye diseases, Head and neck cancer

## Abstract

Patients undergoing intensity-modulated radiotherapy (IMRT) for head and neck cancer may have increased incidence of dry eye disease and the exact mechanism is unclear. The present study aims to assess tear film and meibomian gland (MG) features in patients who received IMRT for head and neck cancer not involving the orbital area. Twenty-seven patients (64.7 ± 9.8 years) and 30 age-matched controls (61.4 ± 11.0 years) underwent a comprehensive dry eye work-up. Compared to the control group, the patients had more lid margin abnormalities, and worse meibum quality. The MG loss, calculated as (tarsal area-MG area)/tarsal area, was higher in the patient group in both the upper (53.0 ± 12.0% vs. 35.1 ± 10.3%, p < 0.001) and lower lids (69.5 ± 12.6% vs. 48.5 ± 12.5%, p < 0.001). In the patient group, more MG loss in the lower lids correlated with worse meibum quality (r = 0.445, p = 0.029). In contrast, there was no significant difference in aqueous tear production level, measured with Schirmer test. Patients treated with IMRT for head and neck cancer seemed to have comparable lacrimal gland function to the controls despite more dry eye symptoms. However, the patients had MG functional and morphological changes, which may present a higher risk for developing dry eye disease.

## Introduction

A well-functioning tear film is essential for protecting the ocular surface from desiccating stress to ensure comfort of the eye, as well as to maintain optimal vision^1,2^. The tear film consists of a lipid layer and an underlying mucoaqueous layer^3^. The lipid layer is formed by meibomian gland (MG) secretion (meibum), the mucin layer is secreted by the goblet cells and the ocular surface epithelium, and the aqueous component is secreted from the lacrimal glands. Exposure of the ocular surface, lacrimal system, and the eyelids to radiation may cause changes in tear production quality and quantity. The loss of homeostasis of the tear film can lead to dry eye disease (DED)^4^.

In the treatment of head and neck cancer (HNC), radiotherapy (RT) can be used alone as the primary treatment modality, in combination with chemotherapy, or as adjuvant therapy following surgical resection. Radiotherapy-induced toxicity can be classified as acute (during or shortly after treatment) or late (> 3 months after completing treatment), and may significantly impact on patients´ quality of life. The most common ocular late effects after RT include radiation keratopathy, cataract, retinopathy, optic neuropathy, and chronic DED^5–9^. Studies have shown that radiation dose and/or dose per fraction are often significant factors in determining the clinical outcomes^10–14^.

Over the years, RT has undergone refinements to improve treatment delivery and outcome. Intensity-modulated radiotherapy (IMRT) is an advanced RT planning technique that uses non-uniform radiation beam intensities to maximize the delivery to the target volume while minimizing the RT dose to the surrounding normal tissues. This allows the prospect of increasing the probability of locoregional disease control while decreasing the complication rate^15^. In HNC treatment, studies have demonstrated that IMRT significantly reduced the incidence and severity of acute and late radiation-induced adverse effects, such as mucositis, xerostomia, radiation-induced blindness, and severe dry eye compared to conventional treatment^15–19^. However, IMRT did not completely alleviate the incidence of DED and the exact mechanism is unclear. The present study aims to evaluate the clinical features of DED in patients who have undergone IMRT for HNC through comprehensive clinical dry eye tests including meibography analysis.

## Methods

### Study participants

This cross sectional, observational study was part of the project investigating the oral and ocular late effects in HNC patients who had received IMRT^20^. The current study included 27 patients with HNC who had completed IMRT treatment at least six months ago, and 30 age-matched healthy controls. The participants underwent a comprehensive evaluation for ocular dryness. The patients and tumor characteristics are summarized in Table 1 and in Supplementary Table A. Tumor staging was according to the 7th edition of the tumor-node-metastasis (TNM) classification of malignant tumors by the International Union Against Cancer (UICC)^21^.

**Table 1:** Patient characteristics.

	Patients (%)
**Sex**	
Male	15 (56%)
Female	12 (44%)
**Original disease locations**	
Oropharyngeal cancer	14 (52%)
Oral cancer	5 (19%)
Parotid gland cancer	5 (19%)
Nasopharyngeal cancer	1 (4%)
Unknown primary, malignant lymph node of the neck	2 (7%)

The patients underwent IMRT for HNC at the Department of Oncology, Oslo University Hospital, Norway. Thirteen patients were treated with primary RT with a total dose of 68–70 Gy, and 14 patients received postoperative RT with a total dose of 56–66 Gy. RT was delivered at 2 Gy per fraction, 5–6 times a week, according to standard treatment guidelines. The RT volume varied according to tumor localization and extension. According to the guidelines, no shield was used to protect the lacrimal gland or the eye, as the treatment volumes were distant from the eye. Concomitant chemotherapy was used for patients aged < 70 years old, as part of primary treatment for Stage III-IV disease, or as part of postoperative treatment if there were involved margins or perinodal infiltration. A total of 11 patients underwent concurrent chemotherapy that consisted of cisplatin or cetuximab given weekly for 3–6 courses.

All participants in the control group (11 males, and 19 females) had a negative history of dry eye/dry mouth complaints, systemic disorders with potential ocular involvement, ocular diseases, previous surgery, and the use of medications that may affect lacrimal and salivary gland secretion.

The Regional Medical Ethical Committee of South-East Norway approved the study (2018/1313). Written informed consent was obtained from all participants, and the study was performed in compliance with the tenets of the Declaration of Helsinki.

### Clinical evaluation

The participants were required not to use topical eye drops within two hours prior to the clinical examination. After completing the Ocular Surface Disease Index (OSDI) questionnaire^22^ (Allergan Inc., Irvine, CA) for assessing subjective symptoms of DED, the participants underwent the following ophthalmic examinations: lower tear meniscus height (TMH) measured with the Keratograph 5 M (OCULUS, Wetzlar, Germany)^23^, tear film break up-time (TFBUT) measurement after instillation of 5 µl of 2% fluorescein sodium, ocular surface fluorescein staining (OSS) of the cornea and bulbar conjunctiva recorded according to the Oxford scoring scheme (range of ocular surface staining: 0–15)^24^, Schirmer I test without topical anesthesia, evaluation of eyelid margin abnormalities and meibomian gland (MG) functionality of the lower eyelids under slit lamp microscopy, and non-contact infrared meibography of both the upper and lower eyelids with Keratograph 5 M after eversion of the eyelids.

Lid margin abnormalities (LMA) were scored as 0 (absent) or 1 (present) for each of the following features: lid margin telangiectasia, lid margin hyperemia, lid margin thickening, and irregular lid margin. A summed score of all four features was recorded as LMA score (range: 0–4). MG functionality was assessed by applying light pressure using cotton tips on the central five MGs of the lower eyelid. Meibum expressibility (ME) was recorded as the number of the MGs with meibum secretion under the pressure. The quality of the meibum that was secreted from each gland was graded as follows: 0, clear; 1, cloudy; 2, cloudy with particles; and 3, toothpaste like. To avoid giving a false low value in cases where many MG orifices were plugged, the meibum quality (MQ) value was averaged with the number of glands that expressed meibum per eyelid. In cases where all MG orifices were plugged, the average MQ per gland was recorded as 3 instead of 0.

**Figure 1:**
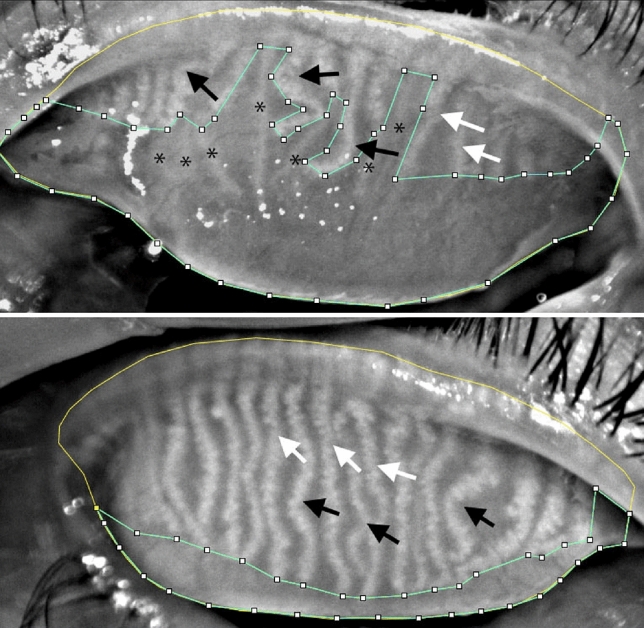
Image J assisted meibomian gland dropout evaluation in one patient (upper) and one subject from the control group (lower). The light green outlined regions represent the dropout area, while yellow outlined regions represent the tarsal plate. The MG loss was 59.9% and 15.3% in the patient and control subject, respectively. The glands with white arrows are examples of distorted glands, whereas the glands with black arrows are examples of tortuous glands. Glands marked with * represent ghost glands which are pale glands with absence of normal meibomian gland architecture.

A masked, experienced observer evaluated the meibography images using computer-assisted analysis with ImageJ software (National Institutes of Health, New York, NY, USA). The MG loss was calculated as follow: (dropout area)/tarsal area, and was assessed separately for the upper and lower eyelids (Fig. 1). The MG loss is presented on a 0–100% scale, where 0% indicates no MG loss, whereas 100% represents complete MG loss. Ghost glands and fluffy areas were included in the dropout area^25^. Additional computerized morphological analyses of the MGs were performed on the upper eyelids only, including the number of all glands, number of distorted glands, and number of tortuous glands, in the whole tarsal plate and in the middle third of the upper lids.

The values of the eyes on the side that received higher radiation dose at the lacrimal gland were used for inter-group comparison. If the radiation dose were the same on both sides, and in the control subjects, the mean values of the two eyes of each subject were used.

The percentage of pathological findings of each clinical test in each group was calculated. The following cut-off values suggested by DEWS II diagnostic report^26^ were used: OSDI questionnaire score ≥ 13, TFBUT < 10 s, and Schirmer I test ≤ 10 mm/5 min. Due to a lack of agreement among the other dry eye tests, OSS ≥ 3, ME < 5, MQ > 0, and LMA score > 0, were adopted as pathological cut-off values in the present study.

### Statistical analysis

Statistical analyses were performed using the commercial software SPSS for Mac, version 25 (IBM, Chicago, IL, USA). Continuous data are expressed as the mean ± standard deviation, and categorical variables are expressed as percentages. The normality of data distribution was evaluated using the Shapiro–Wilk test. According to the normality of data distribution, differences in age between the patients and controls were compared using independent sample t test, while intergroup comparisons of clinical parameters were performed using Mann–Whitney U test. Binomial variables were compared with the χ^2^ test. Correlations between morphological analyses of meibography and other clinical tests, as well as between the total dose of radiation with clinical findings in the patient group, were assessed using Spearman rank correlation analysis. A p-value of < 0.05 was considered significant.

## Results

The mean age of the patients and the controls was 64.7 ± 9.8 years (range, 41–82 years) and 61.4 ± 11.0 years (range, 43–80 years), respectively (p = 0.245). Total RT dose in the patients was 64.9 ± 4.0 Gy. The RT dose to the lacrimal gland on the exposed side of the head was 1.8 ± 4.2 Gy (range 0.3–17.5 Gy). Only two eyes had radiation dose > 10 Gy to the lacrimal gland (15.0 and 17.5 Gy). The mean interval between ocular examination and the last RT fraction was 30.8 ± 15.0 months (range 7.5–65.0 months). The interval was < 12 months in two patients (7.5 and 10.2 months).

### Clinical dry eye tests

**Figure 2:**
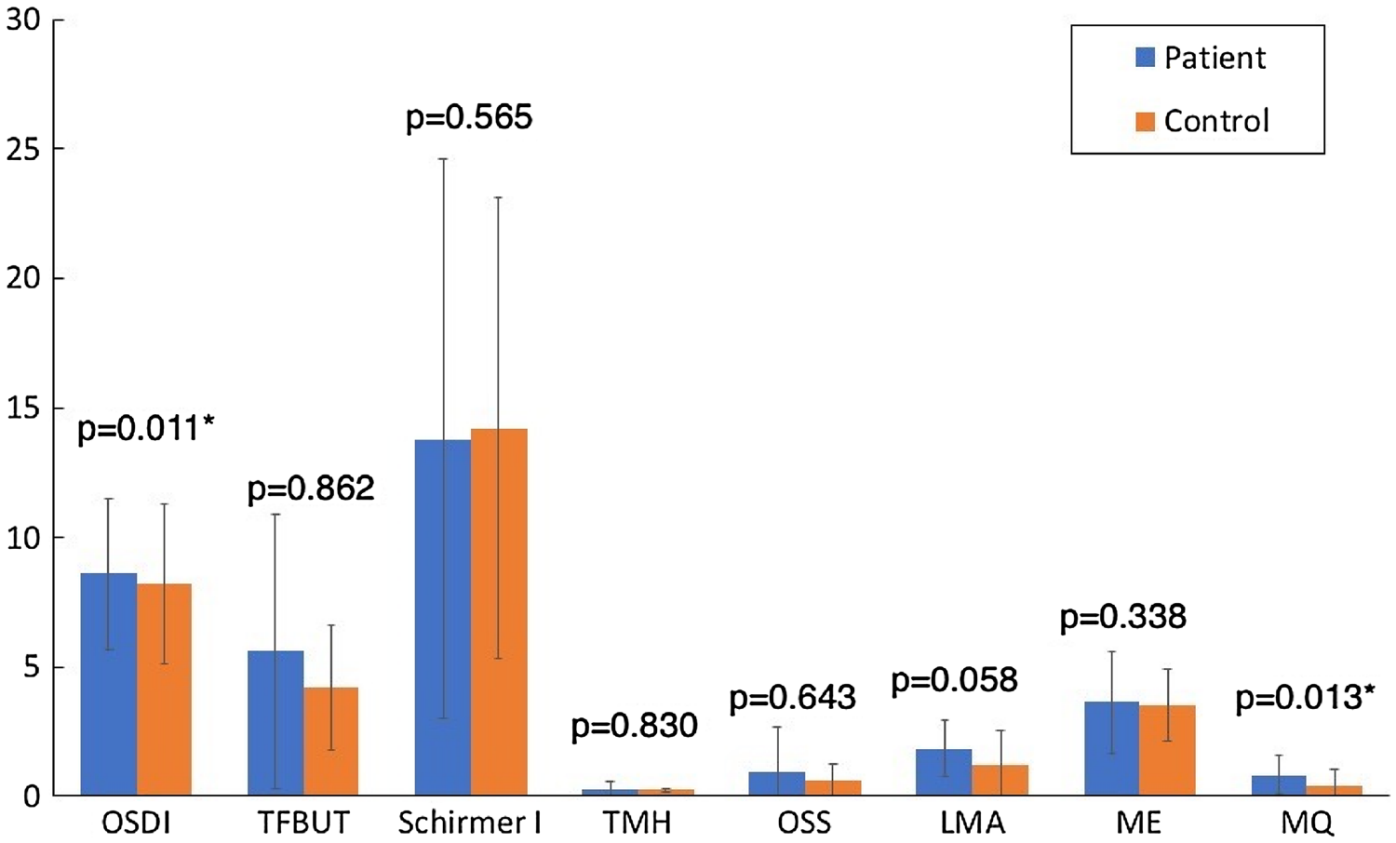
Clinical dry eye tests in patients and controls. *OSDI* Ocular Surface Disease Index questionnaire, *TFBUT* tear film break-up time, *TMH* tear meniscus height, *OSS* ocular surface staining, *LMA* lid margin abnormality, *ME* meibomian gland expressibility, *MQ* meibum quality. *Statistically significant inter-group difference tested with Mann–Whitney U test.

**Figure 3:**
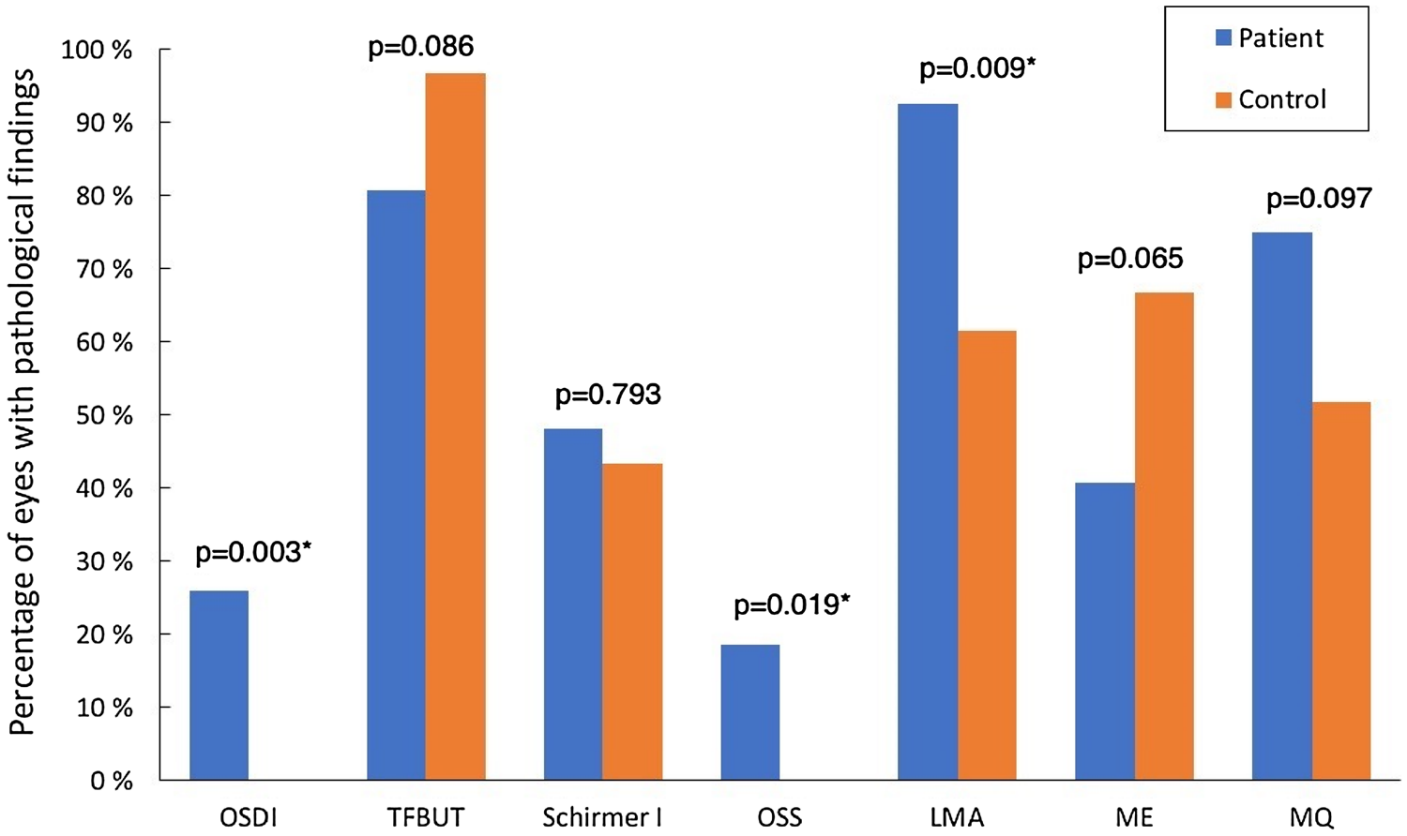
Percentage of abnormal results of the dry eye diagnostic tests obtained in patient and control groups. *OSDI* Ocular Surface Disease Index questionnaire, *TFBUT* tear film break-up time, *OSS* ocular surface staining, *LMA* lid margin abnormality, *ME* meibomian gland expressibility, *MQ* meibum quality. *Statistically significant inter-group differences using the χ^2^ test.

Compared to the control subjects, the patients reported more dry eye complaints, as indicated by the higher OSDI questionnaire score (8.6 ± 8.2 vs. 2.9 ± 3.1, p = 0.011). In addition, they had higher LMA scores (1.9 ± 1.1 vs. 1.3 ± 1.3, p = 0.058) as well as worse MQ (0.8 ± 0.7 vs. 0.4 ± 0.6, p = 0.013) (Fig. 2). Although the mean value of OSDI in the patient group was within the normal range of 12 or below, 25.9% patients demonstrated pathological OSDI of ≥ 13. Further, the percentages of eyes presenting pathological values in the OSS and LMA were also higher in the patient group compared to the controls (OSS: 18.5% vs. 0.00%, p = 0.019; LMA: 92.6% vs. 61.5%, p = 0.009) (Fig. 3). Other clinical dry eye tests did not show statistically significant differences (Figs. 2 and 3).

No statistically significant differences were detected between male and female patients. While comparing the patients who underwent concurrent chemotherapy with those who did not, no statistically significant differences was detected for most of the dry eye tests, except for MQ (0.5 ± 0.5 with chemotherapy vs. 1.1 ± 0.8 without chemotherapy, p = 0.035). There was no significant correlation between total radiation dose, radiation dose to the lacrimal gland, or clinical tests (all, p > 0.05).

### Morphological changes of the meibomian glands

MG loss was higher in the patient group compared to the control group for both the upper (53.0 ± 12.0% vs. 35.1 ± 10.3%, p < 0.001) and lower lids (69.5 ± 12.6% vs. 48.5 ± 12.5%, p < 0.001). The distribution of morphological features of the upper lid MGs was not significantly different between the patients and controls (Table 2).

**Table 2:** Frequency of upper lid meibomian gland features.

Meibomian gland features	Patients	Controls	p-value
**Whole upper lid**			
Total number glands	20.6 ± 3.3	19.1 ± 3.1	0.112
Distorted glands	12.0 ± 3.0	11.8 ± 3.2	0.733
Tortuous glands	7.5 ± 2.8	6.4 ± 2.7	0.188
**Middle third of the upper lid**			
Total number glands	8.2 ± 1.4	7.8 ± 1.3	0.231
Distorted glands	6.5 ± 1.9	6.4 ± 1.8	0.24
Tortuous glands	4.4 ± 1.7	4.0 ± 1.6	0.314

In the patient group, MG loss in the lower eyelid showed a positive correlation with MQ (r = 0.445, p = 0.029). No significant correlation was observed between MG loss and other clinical tests. There was no significant difference in the measured MG morphological features between patients with or without concurrent chemotherapy (p > 0.05 in all comparisons).

## Discussion

The present study shows that around 26% of the patients treated with IMRT for HNC had positive dry eye related complaints. The patients also exhibited more meibomian gland pathology expressed as more LMA, worse MQ, and more MG loss than the controls.

Dry eye disease is a relatively common late effect of RT for HNC involving the periorbital area^11^. For patients who receive high RT doses to treatment volumes near the eye, the onset of DED symptoms is reported to be 9–10 months after treatment^14^. The onset of DED may be caused by radiation damage to several ocular structures, including the lacrimal gland, conjunctival goblet cells, and the MGs. The incidence of DED after RT is considered to be dependent on the total dose of the radiation delivered to the orbital area and dose per fraction. In treatment of orbital lymphoma, Stafford et al.^27^ reported 0% incidence of moderate to severe DED in patients who received a total dose of < 35 Gy. This is in agreement with Letschert and associates^12^ who found no occurrence of sicca syndrome in patients with orbital lymphoma who received a tumor dose < 40 Gy. Some studies found that a dose per fraction of close to or > 2 Gy was a significant risk factor for the incidence of DED^10–14^, and that the incidence of DED increased with an increase in the dose per fraction^11^. The possible synergistic effect of chemotherapy in the development of DED remains anecdotal^11,14^.

Dry eye disease after RT is most commonly attributed to damage to the lacrimal gland acinar cells and secretory abnormalities^9,28^, which would cause reduced aqueous tear production. Parsons et al.^14^ studied the association between severe DED that led to visual loss secondary to corneal opacification, ulceration, or vascularization, and the radiation dose of the lacrimal apparatus. They reported that, in patients who underwent RT for primary extracranial tumors that required irradiation of the eyes or optic nerves, all patients who received doses of ≥ 57 Gy to the lacrimal gland developed severe DED, whereas the incidence in patients who received doses of 30–45 Gy and < 30 Gy were 30% and 0%, respectively. The patients in the present study had cancer that did not involve the area in or around the orbit, and the radiation to the lacrimal glands varied between 0.3 and 17.5 Gy, which was far less than 30 Gy. Accordingly, aqueous tear production measured with the Schirmer I test or TMH did not show significant differences between the patients and the controls. Here, we did not test the lacrimal canal function, but lacrimal canal stenosis after radiation^29^ could theoretically lead to falsely high values in the Schirmer I test or TMH.

Radiation can also cause involutional atrophy of the MGs^30^. However, studies on the influence of RT on MGs are rare^31,32^. MGs secrete lipids that form the superficial layer of the tear film, which help stabilize and prevent evaporation of the tear film. Meibomian gland dysfunction (MGD), a qualitative and/or quantitative change of meibum secretion, is the most common cause of DED. Existing methods for diagnosing MGD include slit-lamp examination of the lid margins, assessment of the MG functionality, and meibography. The incidence of changes in the lid margin, hyposecretion of meibum, and severity of meibomian gland loss are related to age^33,34^. Some studies have shown that sex hormones, especially androgens, can regulate MGs and lipid secretion^35–37^. Woo et al.^31^ investigated the influence of periocular RT for conjunctival or orbital lymphoma on MGs in the lower eyelid. In their study, when all eyes that underwent RT were included, the patients showed worse results for Schirmer test, TFBUT, ME, and more MG loss compared to the age-matched controls. Moreover, the MG loss correlated significantly with ME and total radiation dose. In patients who received a cumulative radiation dose of < 30 Gy, the Schirmer I test did not show a significant difference, but the difference in MG loss remained significant. Similarly, in the current study, the patients had worse MQ and LMA, as well as more MG loss, as compared to the controls. Also, MG loss in the lower eyelid in our patients correlated with MQ. However, we did not find a correlation between the total radiation dose and MG loss. The inconsistency might be attributed to the relatively low number of patients in both studies, varying follow-up times, as well as differences in the two study populations, i.e. age (46.0 ± 16.9 years vs. 64.6 ± 9.6 years in our study), variation of diagnoses, disease sites, cumulative radiation dose (mean 34.7 Gy, 18–110 Gy vs. mean 65.0 Gy, 56–70 Gy). Moreover, the effect of radiation dose on the meibomian gland needs further exploration.

Although the other clinical tests such as the Schirmer I test, TFBUT, ocular surface staining, and ME did not show significant inter-group differences, it does not mean that all patients had normal functional ocular surfaces. Among the patients, over 25% of the patients had OSDI of ≥ 13. Quite a large proportion of the patients exhibited abnormal values in the dry eye tests. In fact, some of the controls also had abnormal values in the clinical dry eye tests, although they did not have dry eye complaints. This is in accordance with the findings of Sullivan et al.^38^ who reported that > 40% of people with clear objective evidence of dry eye disease are asymptomatic. Further, the incidence of DED increases with age. Thus, our controls may not serve as ideal “healthy” controls, but may be more representative of the age-matched normal population. Prospective studies comparing dry eye status before and after IMRT in patients with HNC may better reveal the influence of IMRT on DED.

The chemotherapy agents may also affect the ocular surface, leading to DED and/or alterations in MGs. The current study compared the dry eye and MG features in patients who received IMRT and concomitant chemotherapy with those who received IMRT alone. No statistically significant differences were detected between tests, except that MQ was slightly better in patients who received concomitant chemotherapy. Chemotherapy was used only for patients aged below 70 years. The relatively younger age might explain a better meibomian gland function in this group, as aging is a well-recognized risk factor for meibomian gland dysfunction^39^. In addition, the relatively small sample size might have affected the statistical significance, and a future study involving a larger group of patients is warranted. Moreover, to further study the different impacts of IMRT and chemotherapy on DED and/or alterations in MGs it could be argued that to include a control group of HNC patients treated with chemotherapy or immunotherapy alone may be valuable. However, in the current study such a control group is unsuitable since chemotherapy and immunotherapy alone is only used as palliative treatment^40,41^, whereas patients in this study were treated with curative intent. In future studies the prevalence of DED and/or alterations in MGs could be compared in HNC patients treated with palliative RT and chemotherapy or immunotherapy alone.

In conclusion, patients who were treated with IMRT for HNC not located near the orbital area seem to have comparable lacrimal gland function to the controls. However, we demonstrate functional and morphological changes in the MGs, which may pose a higher risk for the development of DED. Strategies for preventing MGD may reduce the risk of DED in patients in the future.

## Supplementary Information

Supplementary Information.
