# Supplementary material for: Alterations in meibomian glands in patients treated with intensity-modulated radiotherapy for head and neck cancer
Source: Sci Rep. 2021 Nov 17;11:22419. doi: 10.1038/s41598-021-01844-9 (PMC8599465; doi:10.1038/s41598-021-01844-9)
Supplement: Supplementary file 1 — Supplementary Information. [file 41598_2021_1844_MOESM1_ESM.docx]

**Table A. Diagnosis and treatment of study patients.**

| **Study ID** | **Diagnosis** | **TNM-classification** | **Laterality** | **Type radiation** | **Total dose (Gy)** |
| --- | --- | --- | --- | --- | --- |
| B601 | Malignant neoplasm of parotid gland | T2N0M0 | Right | Postoperative | 56 |
| B602 | Malignant neoplasm of parotid gland | T2N2bM0 | Left | Postoperative | 66 |
| B603 | Secondary and unspecified malignant neoplasm of lymph nodes | T0N2aM0 | Both | Postoperative | 60 |
| B604 | Malignant neoplasm of parotid gland | T3N2bM0 | Left | Postoperative | 66 |
| B606 | Malignant neoplasm of tonsillar fossa | T2N2bM0 | Both | Primary | 68 |
| B607 | Malignat neoplasm of border of tongue | T2N1M0 | Both | Postoperative | 60 |
| B608 | Malignant neoplasm of base of tongue | T1N1M0 | Both | Primary | 68 |
| B609 | Malignant neoplasm of parotid gland | T2N0M0 | Right | Postoperative | 66 |
| B610 | Malignant neoplasm of tonsillar fossa | T2N2bM0 | Both | Primary | 70 |
| B611 | Malignant neoplasm of tonsil, unspecified | T2N2bM0 | Right | Postoperative | 66 |
| B612 | Malignant neoplasm of tonsillar fossa | T2N0M0 | Right | Primary | 68 |
| B613 | Malignant neoplasm of tonsil, unspecified | T2N1M0 | Both | Primary | 68 |
| B614 | Malignant neoplasm of tonsillar fossa | T3N2bM0 | Both | Primary | 68 |
| B615 | Malignant neoplasm of other and unspecified parts of mouth | T2N0M0 | Left | Postoperative | 60 |
| B616 | Secondary and unspecified malignant neoplasm of lymph nodes | T2N2bM0 | Both | Primary | 68 |
| B617 | Malignant neoplasm of tonsillar fossa | T1N1M0 | Right | Postoperative | 60 |
| B618 | Malignant neoplasm of parotid gland | T4aN0M0 | Left | Postoperative | 60 |
| B619 | Malignant neoplasm of overlapping sites of oropharynx | T2N1M0 | Both | Primary | 68 |
| B620 | Malignant neoplasm of tonsil, unspecified | T1N1M0 | Left | Postoperative | 60 |
| B621 | Malignat neoplasm of border of tongue | T3N0M0 | Both | Postoperative | 66 |
| B622 | Malignant neoplasm of lower gum | T4N1M0 | Left | Postoperative | 60 |
| B623 | Malignant neoplasm of base of tongue | T2N2cM0 | Both | Primary | 68 |
| B624 | Malignant neoplasm of tonsillar fossa | T2N0M0 | Left | Primary | 68 |
| B626 | Malignant neoplasm of lateral wall of nasopharynx | T2N2bM0 | Both | Primary | 68 |
| B628 | Malignant neoplasm of tonsillar fossa | T4N1M0 | Both | Primary | 68 |
| B629 | Malignant neoplasm of tonsillar fossa | T3N1M0 | Both | Primary | 68 |
| B630 | Malignant neoplasm of lower gum | T4aN2bM0 | Right | Postoperative | 60 |
